# Macronutrients Intake and Stomach Cancer Risk in Iran: A Hospital-based Case-Control Study

**DOI:** 10.34172/jrhs.2021.38

**Published:** 2021-01-12

**Authors:** Fatemeh Toorang, Bahareh Sasanfar, Azita Hekmatdoost, Saba Narmcheshm, Maryam Hadji, Soraiya Ebrahimpour-Koujan, Neda Amini, Kazem Zendehdel

**Affiliations:** ^1^Department of Community Nutrition, School of Nutritional Sciences and Dietetics, Tehran University of Medical Sciences, Tehran, Iran; ^2^Cancer Research Center, Cancer Institute of Iran, Tehran University of Medical Science, Tehran, Iran; ^3^Nutrition and Food Security Research Center, Shahid Sadoughi University of Medical Sciences, Yazd, Iran; ^4^Department of Nutrition, School of Public Health, Shahid Sadoughi University of Medical Sciences, Yazd, Iran; ^5^Department of Clinical Nutrition, Faculty of Nutrition and Food Technology, National Research Institute of Nutrition and Food Technology, Shahid Beheshti University of Medical Sciences and Health Services, Tehran, Iran; ^6^Faculty of Social Sciences, Tampere University, Tampere, Finland; ^7^Department of Surgery, Sinai Hospital of Baltimore, Baltimore, USA; ^8^Department of Surgery, Johns Hopkins Hospital, Baltimore, USA; ^9^Cancer Biology Research Center, Cancer Institute of Iran, Tehran University of Medical Science, Tehran, Iran

**Keywords:** Stomach Cancer, Macronutrient Intakes, Case-Control Study

## Abstract

**Background:** Stomach cancer (SC) is one of the most common cancers in the world. Dietary risk factors of SC are not fully understood. This study aimed to investigate the association between macronutrient intakes and the risk of SC.

**Study design:** A hospital-based case-control study.

**Methods:** The data were obtained from a hospital-based case-control study conducted at the Cancer Institute of Iran from 2010 to 2012. Patients were 40 years or older and were diagnosed with SC in less than one year with no history of any cancers. On the other hand, the controls were healthy subjects who were caregivers or visitors of the patients. Demographic characteristics were collected using a structured questionnaire through face to face interviews by trained interviewers. Dietary data were obtained using a validated Diet History Questionnaire. The age and gender-adjusted odds ratios (ORs), as well as the adjusted ORs of age, gender, energy, education, smoking, and body mass index, were reported for continuous and tertiles of intakes.

**Results:** Totally, 207 SC patients and 217 controls participated in this study. In the full adjusted model, after comparing the highest tertiles to the lowest ones, the intake of sucrose (OR: 2.94; 95% CI: 1.66-5.19; P-trend<0.001), protein (OR: 2.04; 95% CI: 1.17-3.55; P-trend=0.011), cholesterol (OR: 2.22; 95% CI: 1.28-3.85; P-trend=0.005), and percent of calories from protein (OR: 3.09; 95% CI: 1.69-5.61; P-trend<1.001) showed a positive significant association with SC. Moreover, a significantly negative association was found between the percent of calories obtained from carbohydrates and SC (OR: 0. 57; 95% CI: 0.33-0.98; P-trend=0.015).

**Conclusion:** The findings in this study showed that macronutrient intakes might be associated with the etiology of SC in Iran.

## Introduction

Stomach cancer (SC) is one of the most common cancers in the world. Although its incidence is decreasing in most developed countries, it is known as the fifth common cancer and the third cause of cancer death worldwide^1^. Its incidence rate is double in males and increases by aging in both genders ^2^. The SC incidence is high in Iran and is the most common cancer in males. The Age Standard Rates of SC were estimated at 20.6 and 9.7 per 100,000 populations in males and females, respectively ^3^. Approximately, 80% of patients are diagnosed in advanced stages and do not benefit from therapeutic strategies ^4,5^. Therefore, prevention strategies could play an important role in SC control.

Several risk factors have been studied concerning stomach cancer. *Helicobacter Pylori* infection has been recognized as the main risk factor in this regard, along with genetic factors, gender, tobacco smoking, heavy alcohol consumption, and body fatness ^6-8^. Furthermore, dietary factors are considered to have an important role in the etiology of SC. Several studies have investigated the association between dietary factors and SC leading to controversial results. Some studies found a direct association of risk of SC with the intake of protein ^9-11^, fat^12^, saturated fat^13^, and carbohydrate. However, other studies reported negative or null association with protein ^12^, as well as monounsaturated and polyunsaturated fats ^14,15^. The World Cancer Research Fund International in its latest report recognized obesity and foods preserved by salt as probable nutritional risk factors for SC ^16^. Additionally, it declared there is limited evidence on the association between SC and other nutritional factors, followed by no conclusion on the effect of nutrients intake on SC. Given the inconsistent results of previous studies, this study aimed to investigate the association of SC with energy and macronutrients intake in Iran.

## Methods

The data were obtained from a hospital-based case-control study conducted at the Cancer Institute of Iran from 2010 to 2012. It is noteworthy to mention that this institute is the most comprehensive cancer center located in Tehran, capital city of Iran that admits cancer patients from all parts of this country^17^. The SC is rare in people younger than 40 years of age; and if it occurs, it is mainly due to genetic factors. Therefore, this study recruited 210 patients who were 40 years old or over and were diagnosed with SC in less than one year with no history of any cancers. An experienced pathologist assessed gastroscopic or surgical biopsy of the patients.

 Accordingly, the patients with histologically confirmed stomach cancer (defined by the Second Edition of the International Classification of Diseases for Oncology; ICD-c16) were included in this study. The controls consisted of 223 healthy relatives of patients who were referred to the hospital to visit or take care of their relatives. The healthy controls were enrolled since the dietary intakes of the patients were usually changed due to their disease. The controls were matched with the cases regarding number, residential place, and age (±5 years). As mentioned, the inclusion criteria were 1) age of older than 40 years, 2) lack of no chronic diseases (except GS in patients), and 3) willingness to participate in the study. On the other hand, the pregnant or breastfeeding female patients, as well as those who were diagnosed with cancer more than a year or being affected by SC as a second cancer were excluded from the study. Written informed consent was obtained from all participants after oral and face to face explanation of the study protocol and objectives. The study protocol was approved by the Ethical Committee of Tehran University of Medical Sciences, Tehran, Iran (no.17198).

Demographic characteristics of the participants were collected through a structured questionnaire by a face to face interview conducted by trained interviewers. Following that, trained nutritionists conducted face to face interviews to complete the Diet History Questionnaire (DHQ). This questionnaire is a kind of Food Frequency Questionnaire (FFQ), which developed by the American Cancer Institute to study the nutritional factors associated with cancers ^18^. This questionnaire was previously modified according to Iranian food habits, and its validity was confirmed in a previously conducted study ^19^. This questionnaire includes 146 questions related to the consumption of foods and Iranian mixed dishes during the last 12 months. The cases were asked to recall their intake before the appearance of disease symptoms (one year before the diagnosis).

 Moreover, the interviewers reminded the patients several times through the interviews that they should answer the questions based on their dietary habits before the onset of the cancer symptoms. They used Iranian portions of food to help subjects remember their food intake properly. If it seems that subjects are over- or under-reporting, the interviewer asked how much food was usually served on that occasion and how many people ate these portions of foods to help them recall more preciously. Since bread is the staple food of Iranian population, interviewers initially asked how much bread was eaten in a day. Subsequently, they asked about the consumption of different kinds of bread, and if there were wide range of differences, they attempted to help them recall the correct amount of consumption. The DHQ contained questions about dietary supplement consumption in order to dismiss people who take supplements. However, it was not common in our study population, and no one was dismissed by this reason. An experienced nutritionist checked all questionnaires, and the missing data were obtained through telephone calls.

The data obtained from the DHQ were converted to gram/day in separated programs made by the authors in STATA statistical software. Daily food intakes were inverted to energy and nutrient intakes using a food composition table. As the Iranian food composition table covers only raw foods and limited nutrients ^20^, McCance and Widdowson's tables of Food composition ^21,22^ were utilized and supplemented by Iranian ones ^20^ for some special foods that are consumed in Iran. The procedure is explained in detail elsewhere ^19^.

 The Body Mass Index (BMI) was calculated as weight in kilograms divided by height in squared meters. As SC affects the weight of patients, they were requested to recall their usual weights rather than the recent weight. Smoking status was defined through self-reporting and classified by ever smoking and non-smoking at any time in the last year.

###  Statistical analysis 

The sample size was estimated in Stata software (version14) using a power of 90%, a significance level of 0.05, and an OR of 1.8 to investigate the association between macronutrient intake and risk of SC. Accordingly, the sample size was estimated at a minimum of 192 cases to study this association in the Iranian population ^9,23-25^. As one gram of nutrient intake could not make any significant differences to cancer risk, it was suggested to the group subjects based on their classification of intakes, followed by a comparison between the higher and lower intakes ^26^. Therefore, the multivariate unconditional logistic regression model was employed to estimate Odds Ratios (OR) and 95% confidence intervals of SC according to raw and tertiles of energy and macronutrient intakes. Furthermore, the residual method explained by W. Willet was used to adjust for energy intakes^27,28^. The model A was adjusted for age, gender and energy and the model B was further adjusted for education, smoking and BMI. The P for trend were estimated putting continues form of nutrients intake in models.

## Results

After withdrawing the over- and under-reporting subjects, 207 newly diagnosed SC patients and 217 controls were included in this study. The response rates were 95% and 70% among cases and controls, respectively. According to the results, the SC patients were less educated (*P*<0.01) and older (64.3 ±12.2 vs. 53.9 ±11.6; *P*<0.001) than the controls. Moreover, they consumed less energy (2432.9 ±1224.8 vs. 2992.3 ±1266.2; *P*<0.001), compared to the controls (Table 1).

**Table 1:** Descriptive characteristic of the subjects in this case-control study to evaluate the association of gastric cancer with dietary factors in the Cancer Institute of Iran (2010-2012)

**V ariables**	**Cases (n=207)**		**Controls (n=217)**		***P*** **-value**
**Categorical **	**Number**	**Percent**	**Number**	**Percent**	
Gender					0.92
Males	132	61	158	76	
Females	146	39	59	24	
Education					0.001
Illiterate	137	66	75	33	
Primary school	41	20	103	47	
High school	20	10	22	10	
College	9	4	20	9	
Smoking					0.204
No	76	37	67	31	
Yes	131	64	150	69	
**Continuous**	**Mean**	**SD**	**Mean**	**SD**	***P*** **-value**
Age (years)	64.3	12.3	53.9	11.6	0.001
Body mass index (kg/m^2^)	27.5	15.9	25.4	4.6	0.067
Energy (kcal/day)^a^	2432.9	1224.8	2992.3	1266.2	0.001

^a^ Obtained from chi-square test for categorical variables and independent student's t-test for continuous variables

Table 2 tabulates the intakes of macronutrients in the SC patients and controls. As can be seen, the SC showed a positive association with sucrose (OR: 1.03; 95% CI: 1.01, 1.04), total fat (OR: 1.02; 95% CI: 1.01, 1.03), saturated fats (OR: 1.03; 95% CI: 1.01, 1.05), and percent of calorie from protein (OR: 1.12; 95% CI: 1.05-1.19) in model A. After adjusting for more variables in model B, a positive association was found between SC and sucrose (OR, 1.03; 95% CI: 1.02-1.05), saturated fats (OR: 1.03; 95% CI: 1.01-1.05), and percent of calorie from protein (OR: 1.11; 95% CI: 1.04-1.18). Moreover, the protective effects were found for the percent of calories from carbohydrates (OR: 0.97; 95% CI: 0.94-0.99) in model A. It should be noted that the adjustments for more variables in model B led to no changes in the association (Table 2).

Table 3 tabulates the ORs and the corresponding 95% CIs of macronutrient intakes for second and third tertiles versus the first one. Positive associations with increasing intakes were evidenced in sucrose (P-trend>0.001), protein (P-trend=0.005), saturated fatty acids (P-trend=0.048), cholesterol (P-trend=0.003), and percent of calorie from protein (P-trend<0.001) in model A that were adjusted only for age, gender, and energy intake. Further adjustment for education, smoking, and BMI in model B led to no notable changes in the associations. In this model, after comparing the highest to the lowest, the intake of sucrose (OR: 2.94; 95% CI: 1.66-5.19; P-trend<0.001), protein (OR: 2.04; 95% CI: 1.17-3.55; P-trend=0.011), cholesterol (OR, 2.22; 95% CI: 1.28-3.85; P-trend=0.005), and percent of calories from protein (OR: 3.09; 95% CI: 1.69-5.61; P-trend<0.001) showed a positive significant association with SC. However, a negative association of SC was shown with the percent of calories from carbohydrates (P-trend=0.02) in model A, which remained significant in model B. Furthermore, a significantly negative association was found between SC and the percentage of calories from carbohydrate in model B (OR: 0. 57; 95% CI: 0.33-0.98; P-trend=0.015).

**Table 2:** Energy and macronutrient intakes and risk of gastric cancer in this case-control study in the Cancer Institute of Iran (2010-2012)

**Variables**	**Cases (n=207)**		**Controls (n=217)**		**Crude** **OR (95% CI)**	**Partial adjusted** **OR (95% CI)** ^a^	**Full adjusted** **OR (95% CI)** ^b^
	**Mean**	**SD**	**Mean**	**SD**			
Carbohydrate(g/day)	324.7	180.8	409.4	184.3	0.99 (0.99, 1.00)	0.99 (0.99, 1.00)	0.99 (0.99, 1.00)
Fiber (g/day)	18.1	9.7	22.8	10.1	0.95 (0.93, 0.97)	0.98 (0.95, 1.01)	0.99 (0.95, 1.02)
Sucrose(g/day)	33.0	19.6	32.1	17.9	1.00 (0.99, 1.01)	1.03 (1.01, 1.04)	1.03 (1.02, 1.05)
Total protein (g/day)	105.5	44.3	115.8	48.3	0.99 (0.99, 0.99)	1.01 (1.00, 1.02)	1.01 (0.99, 1.02)
Animal protein (g/day)	89.1	41.7	86.2	39.1	1.00 (0.99, 1.01)	0.99 (0.99, 1.00)	1.00 (0.99, 1.01)
Vegetable protein (g/day)	33.8	20.3	39.2	22.3	0.97 (0.96, 0.98)	0.99 (0.99, 1.01)	0.99 (0.98, 1.01)
Total fat (g/day)^3^	84.2	51.3	10.6	60.0	0.99 (0.99, 0.99)	1.02 (1.01, 1.03)	1.00 (0.99, 1.01)
Animal fat (g/day)	43.8	29.0	42.1	23.1	1.00 (0.99, 1.01)	1.00 (0.99, 1.01)	1.00 (0.99-1.01)
Vegetable fat (g/day)	52.2	45.8	61.9	52.3	0.99 (0.98, 0.99)	0.99 (0.99, 1.00)	0.99 (0.99-1.00)
Saturated Fatty Acids(g/day)	25.0	17.9	27.2	14.1	0.99 (0.98, 1.01)	1.03 (1.01, 1.05)	1.03 (1.01, 1.05)
Monounsaturated Fatty Acids (g/day)	21.7	13.1	26.4	14.2	0.98 (0.98, 0.99)	1.02 (0.99, 1.05)	1.03 (0.99, 1.06)
Polyunsaturated Fatty Acids (g/day)	25.7	22.7	39.9	31.2	0.98 (0.97, 0.99)	0.99 (0.98, 1.00)	0.99 (0.98, 1.00)
Cholesterol (mg/day)	249.9	146.3	246.5	133.6	1.00 (0.99, 1.00)	1.00 (1.00, 1.00)	1.00 (1.00, 1.01)
Percent of calorie from carbohydrates	53.4	7.9	55.0	8.8	0.97 (0.95, 0.96)	0.97 (0.94, 0.99)	0.97 (0.94, 0.99)
Percent of calorie from proteins	18.3	4.1	15.9	3.8	1.17 (1.11, 1.23)	1.12 (1.05, 1.19)	1.11 (1.04, 1.18)
Percent of calorie from fats	30.7	7.5	31.3	9.3	0.99 (0.97, 1.01)	1.01 (0.98, 1.04)	1.01 (0.99, 1.04)

^a^ Adjusted for age, gender, and energy b^b^Adjusted for age, gender, energy, education, smoking, and body mass index

## Discussion

 The obtained results from this study showed a positive and dose-response association of the risk of SC with dietary intake of sucrose, protein, cholesterol, and percentage of calories from protein in the Iranian population; however, the percentage of calories from carbohydrates showed a negative association in this regard.

In the present study, simple carbohydrate intake was associated with a higher risk of SC; moreover, the percent of calories from carbohydrates was associated with a lower risk of SC. However, no significant association was found between total carbohydrate intake and risk of SC. Previous studies have reported controversial results about the association between dietary intakes and SC risk ^19-24^. A pooled analysis in the USA showed no association between sugar or total carbohydrate intakes and SC ^29^. On the other hand, other studies in American ^10^ and Italian population ^9^ showed an inverse association between the intake of carbohydrates and SC. A recent meta-analysis has revealed no significant association between dietary carbohydrate intake and risk of SC, except for the Asian population ^30^.

**Table 3:** Odds ratios for gastric cancer risk and 95% confidence intervals according to the tertiles of intake of energy and macronutrients in this case-control study in the Cancer Institute of Iran (2010-2012)

**Nutrient/ tertiles of intake**	**Crude** **OR (95% CI)**	***P*** **-trend**	**Partial adjusted** **OR (95% CI)** ^a^	***P*** **-trend**	**Full adjusted** **OR (95% CI)** ^b^	***P*** **-trend**
Carbohydrate		0.380		0.094		0.072
First	1.00		1.00		1.00	
Second	1.63 (1.02, 2.59)		1.31 (0.77, 2.21)		1.29 (0.75, 2.24)	
Third	0.81 (0.51, 1.29)		0.64 (0.38, 1.08)		0.61 (0.35, 1.06)	
Fiber		0.110		0.786		0.757
First	1.00		1.00		1.00	
Second	1.17 (0.73, 1.85)		1.13 (0.67, 1.88)		1.14 (0.66, 1.95)	
Third	0.68 (0.43,1.09)		0.93 (0.55, 1.56)		1.09 (0.63, 1.90)	
Sucrose		0.001		0.001		0.001
First	1.00		1.00		1.00	
Second	1.89 (1.18, 3.04)		1.67 (0.97, 2.79)		1.59 (0.92, 2.75)	
Third	2.73 (1.96, 4.40)		2.50 (1.48, 4.23)		2.94 (1.66, 5.19)	
Protein		0.001		0.005		0.011
First	1.00		1.00		1.00	
Second	1.78 (1.12, 2.86)		1.47 (0.87, 2.47)		1.40 (0.81, 2.42)	
Third	2.43 (1.51, 3.9)		2.11 (1.25, 3.56)		2.04 (1.17, 3.55)	
Animal protein		0.380		0.390		0.687
First	1.00		1.00		1.00	
Second	0.99 (0.62, 1.56)		0.98 (0.58, 1.64)		0.98 (0.57, 1.69)	
Third	1.23 (0.78, 1.95)		0.78 (0.48, 1.34)		0.89 (0.52, 1.54)	
Vegetable protein		0.001		0.774		0.409
First	1.00		1.00		1.00	
Second	0.24 (0.15, 0.39)		1.02 (0.59, 1.75)		0.89 (0.50, 1.57)	
Third	0.22 (0.13, 0.36)		0.93 (0.55, 1.58)		0.79 (0.45, 1.39)	
Total fat		0.380		0.782		0.520
First	1.00		1.00		1.00	
Second	1.93 (1.21, 3.09)		1.61 (0.96, 2.69)		1.71 (1.00, 2.93)	
Third	0.81 (0.5, 1.29)		1.06 (0.63, 1.79)		1.18 (0.68, 2.04)	
Animal fat		0.180		0.854		0.669
First	1.00		1.00		1.00	
Second	0.61 (0.39, 0.98)		1.00 (0.59, 1.68)		0.93 (0.54, 1.60)	
Third	0.73 (0.46, 1.15)		0.95 (0.57, 1.59)		0.89 (0.52, 1.53)	
Vegetable fat		0.001		0.214		0.108
First	1.00		1.00		1.00	
Second	0.7 (0.44, 1.12)		1.04 (0.61, 1.75)		0.99 (0.57, 1.72)	
Third	0.34 (0.21, 0.56)		0.73 (0.43, 1.22)		0.64 (0.37, 1.12)	
Saturated fatty acids		0.040		0.048		0.055
First	1.00		1.00		1.00	
Second	1.72 (1.08, 2.75)		1.98 (1.17, 3.37)		2.21 (1.26, 3.87)	
Third	1.63 (1.02, 2.59)		1.71 (1.01, 2.89)		1.73 (0.99, 3.00)	
Monounsaturated fatty acids		0.860		0.233		0.107
First	1.00		1.00		1.00	
Second	1.77 (1.11, 2.82)		1.76 (1.05, 2.95)		1.89 (1.09, 3.26)	
Third	1.04 (0.65, 1.66)		1.36 (0.81, 2.29)		1.56 (0.90, 2.68)	
Polyunsaturated fatty acids		0.010		0.204		0.448
First	1.00		1.00		1.00	
Second	1.23 (0.78, 1.96)		1.17 (0.71, 1.95)		1.23 (0.72, 2.09)	
Third	0.54 (0.34, 0.87)		0.71 (0.42, 1.19)		0.79 (0.46, 1.38)	
Cholesterol		0.001		0.003		0.005
First	1.00		1.00		1.00	
Second	2.12 (1.32, 3.39)		1.77 (1.05, 2.98)		1.88 (1.09, 3.25)	
Third	2.44 (1.52, 3.92)		2.23 (1.32, 3.77)		2.22 (1.28, 3.85)	
Percent of calorie from carbohydrates		0.070		0.020		0.015
First	1.00		1.00		1.00	
Second	1.04 (0.66, 1.66)		1.04 (0.62, 1.67)		1.05 (0.60, 1.82)	
Third	0.65 (0.41, 1.03)		0.59 (0.35, 1.01)		0.57 (0.33, 0.98)	
Percent of calorie from proteins		0.001		0.001		0.001
First	1.00		1.00		1.00	
Second	1.99 (1.23, 3.24)		1.45 (0.85, 2.47)		1.39 (0.79, 2.44)	
Third	5.05 (3.06, 8.32)		3.39 (1.92, 5.96)		3.09 (1.69, 5.61)	
Percent of calorie from fat		0.263		0.696		0.444
First	1.00		1.00		1.00	
Second	1.34 (0.84, 2.13)		1.22 (0.74, 2.05)		1.36 (0.79, 2.33)	
Third	1.34 (0.84, 2.13)		0.95 (0.56, 1.61)		1.06 (0.61, 1.83)	

^a^ Adjusted for age, gender, and energy b^b^Adjusted for age, gender, energy, education, smoking, and body mass index

High dietary intake of sugar increases blood glucose and insulin ^31^ can contribute to gastric carcinogenesis by the activation of inflammatory, oxidative, and proliferative pathways ^32,33^. Hyperglycemia might increase the production of reactive oxygen species resulting in DNA damage and raising vascular endothelial growth factor expression associated with vascularity and metastasis of tumor ^34^. On the other hand, hyperglycemia induces insulin secretion from Langerhans islets in the pancreas. Insulin acts as a cancer-promoting agent that could promote the bioavailability of insulin-like growth factor (IGF)-1 by the inhibition of IGF-binding protein production ^35,36^. The IGF-1 stimulates cell proliferation and inhibits apoptosis through a series of downstream pathways activated by phosphoinositide3-kinase/protein kinase B and Ras/mitogen-activated protein kinase pathways ^36,37^. On the other hand, total carbohydrate intake is associated with a higher intake of fruit and vegetables and a lower intake of protein and fat which could lower the risk of SC.

The results of this study also demonstrated that a higher intake of protein and a higher percent of calories from protein were associated with a higher risk of SC. Palli et al. suggested that SC was associated with protein intake in Italy^9^, which was consistent with the results of this study. Moreover, another study in the USA showed a positive association between SC and total intake of protein or intake of protein from animal sources^10^. However, in a report by the World Cancer Research Fund and American Institute for Cancer Research in 2016, all investigations on SC and nutrient intakes reached unconvincing results about protein, carbohydrate, and fat intakes ^16^.

The mechanism of the effect of protein restriction on cancer prevention is not fully understood. A decrease in protein intake affects cellular metabolism significantly. Moreover, it changes the energy and amino acids metabolisms, autophagy, immune responses, inflammation, and signaling pathways. It is clear that all these modifications could alter cancer risk. Moreover, protein restriction could modify gut microbiota, which is considered a new way to control cancer ^38^. Cholesterol intake showed a positive association with the risk of SC in this study, which supports the findings of several studies that reported the association between intake of cholesterol and risk of SC ^11,23^; however, there are also conflicting results in other studies ^25,39^. Although the mechanisms are not clear, high cholesterol intake is associated with alterations in apo-lipoproteins and lipids, which may contribute to inflammation ^40^.

Regarding the strength of the present study, one can name the high rates of participation, same socioeconomic status of patients and controls, as well as the use of a validated DHQ. Since the participants were recruited from all parts of the country, the results could be generalized to the whole population across Iran. Moreover, since SC is most prevalent in low- and middle-income countries the risk factors of which are similar to those in Iran, the results could be generalized to these countries^41^. However, since all epidemiological studies apply FFQ, the misclassification of the study participants was unavoidable based on their dietary intakes ^26^. Although several confounders were under control in this study, the possibility of residual confounding variables could not be excluded. Given the case-control design of the study, the inherent limitations of recall and selection bias should also be considered in this study^42^.

## Conclusion

 This study indicated a potential role of some macronutrients intake in the risk of SC, including intake of sucrose, cholesterol, and protein. Further research with a larger sample size is required to confirm these findings and evaluate the above findings in the cardia and non-cardia SC exclusively.

## Acknowledgements

 The authors are grateful to all participants who responded to the questions patiently. Moreover, this study was supported by nurses in the cancer institute of Iran.

## Conflict of interest

 There is no potential conflict of interest.

## Funding

 This study was financially supported by the Cancer Research Center in Tehran University of Medical Sciences, Tehran, Iran (no.17198).

## Contribution of authors

 FT and KZ designed the study, BS and MH supervised data collection and cleaning. FT analyzed the data and wrote the draft under the supervision of KZ and AH. All authors reviewed the final version of the manuscript.

## Ethical consideration

 Written informed consent was taken from all participants after a face-to-face explanation of the study protocol. This study was approved by the Ethics Committee of Tehran University of Medical Sciences, Tehran, Iran (no.17198).

## Highlights

- Stomach cancer is known as the fourth-common cancer in males.
- Unhealthy dietary intake is a modifiable risk factor for gastric cancer.
- Sucrose, cholesterol, and protein intakes could be associated with increased risk of SC
